# Malaria is an uncommon cause of adult sepsis in south-western Uganda

**DOI:** 10.1186/1475-2875-12-146

**Published:** 2013-05-01

**Authors:** Mary A Auma, Mark J Siedner, Dan Nyehangane, Aisha Nalusaji, Martha Nakaye, Juliet Mwanga-Amumpaire, Rose Muhindo, L Anthony Wilson, Yap Boum, Christopher C Moore

**Affiliations:** 1 Faculty of Medicine, Mbarara University of Science and Technology, Mbarara, Uganda; 2 Division of Infectious Diseases, Massachusetts General Hospital, Harvard Medical School, Boston, MA, USA; 3 Epicentre Mbarara Research Base, Mbarara, Uganda; 4 Department of Microbiology, Mbarara University of Science and Technology, Mbarara, Uganda; 5 Department of Medicine, Division of Infectious Diseases and International Health, University of Virginia, Charlottesville, VA, USA

**Keywords:** Sepsis, Uganda, Africa, Malaria, Adult

## Abstract

**Background:**

Malaria is often considered a cause of adult sepsis in malaria endemic areas. However, diagnostic limitations can make distinction between malaria and other infections challenging. Therefore, the objective of this study was to determine the relative contribution of malaria to adult sepsis in south-western Uganda.

**Methods:**

Adult patients with sepsis were enrolled at the Mbarara Regional Referral Hospital between February and May 2012. Sepsis was defined as infection plus ≥2 of the following: axillary temperature >37.5°C or <35.5°C, heart rate >90 or respiratory rate >20. Severe sepsis was defined as sepsis plus organ dysfunction (blood lactate >4 mmol/L, confusion, or a systolic blood pressure <90 mmHg). Sociodemographic, clinical and laboratory data, including malaria PCR and rapid diagnostic tests, as well as acid fast bacteria sputum smears and blood cultures were collected. Patients were followed until in-patient death or discharge. The primary outcome of interest was the cause of sepsis. Multivariable logistic regression was performed to assess predictors of mortality.

**Results:**

Enrollment included 216 participants who were 51% female with a median age of 32 years (IQR 27–43 years). Of these, 122 (56%) subjects were HIV-seropositive of whom 75 (66%) had a CD4+ T cell count <100 cells/μL. The prevalence of malaria was 4% (six with *Plasmodium falciparum*, two with *Plasmodium vivax*). Bacteraemia was identified in 41 (19%) patients. In-hospital mortality was 19% (n = 42). In multivariable regression analysis, Glasgow Coma Score <9 (IRR 4.81, 95% CI 1.80-12.8) and severe sepsis (IRR, 2.07, 95% CI 1.03-4.14), but no specific diagnoses were statistically associated with in-hospital mortality.

**Conclusion:**

Malaria was an uncommon cause of adult sepsis in a regional referral hospital in south-western Uganda. In this setting, a thorough evaluation for alternate causes of disease in patients presenting with sepsis is recommended.

## Background

Severe sepsis is an important cause of death in sub-Saharan Africa (SSA) where the global burden of lethal infectious diseases is highest [1]. Due to resource limitations, including lack of investigative laboratory capacity, the aetiology of infections causing sepsis in this region is frequently unknown leading to empirical antimicrobial therapy. In malaria-endemic regions this frequently includes anti-malarial drugs. Although the majority of malaria rests with children and pregnant women, prevalence can also be high among non-pregnant adult populations, particularly in those infected by HIV [2-6]. One recent report suggested that the prevalence of malaria in adults is higher than previously thought [7]. However, incidental parasitaemia can occur in asymptomatic patients so the contribution of parasitaemia to the course of severe sepsis can be difficult to discern. Furthermore, malaria is frequently over-diagnosed in areas such as Uganda with limited laboratory infrastructure. This can lead to over-treatment and development of resistance to antiparasitic medications [8,9].

The time to the initiation of appropriate antimicrobial therapy is an important determinant of the outcome from severe sepsis [10]. On the other hand, indiscriminate use of antimicrobial therapy leads inexorably to antimicrobial resistance. Therefore, it is critical to determine the microbiological cause of severe sepsis and to focus antimicrobial therapy according to microbiological test results. In malaria-endemic settings, a better understanding of the contribution of malaria to severe sepsis would allow tailored empirical antimicrobial therapy and avoid over- or under-treatment of malaria. Therefore, the objective of this study was to determine the contribution of malaria to sepsis in adults admitted to a regional referral hospital in south-western Uganda. Because rapid diagnostic tests (RDTs) have not been studied in adults with sepsis, a secondary aim was to determine the sensitivity and specificity of bedside malaria tests compared to a polymerase chain reaction (PCR) confirmed malaria diagnosis in the setting of adult sepsis.

## Methods

### Site description

The study was conducted on the medical ward of Mbarara Regional Referral Hospital (MRRH), which is the teaching hospital for the Faculty of Medicine at the Mbarara University of Science and Technology (MUST) and serves a largely rural population of 1.2-2.5 million people from surrounding districts in south-western Uganda. Mbarara District has an average malaria prevalence of 4% in urban areas and 23% in rural areas. The climate is tropical with a bimodal rainfall pattern in September-January and March-May, and an average precipitation of 1,200 mm per annum [11].

### Study population and definitions

Adult patients (age ≥18 years) admitted to the medical ward during the study period from February-May 2012 were screened consecutively during the hours of 08.00-0.00. Patients were included if they met criteria for sepsis which was defined by clinical suspicion of infection and ≥2 of the following: axillary temperature >37.5°C or <35.5°C, heart rate >90 beats/min, and respiratory rate >20 breaths/min. White blood cell concentration (WBC) was not included in the enrolment criteria due to lack of immediate availability of results. Severe sepsis was diagnosed in patients with evidence of end-organ dysfunction (blood lactate >4 mmol/L, confusion, or a systolic blood pressure <90 mmHg) [5]. Patients were excluded if they required triage to a surgical or obstetrics and gynaecology ward. Patients or their attendant (an accompanying family member or friend) provided informed consent prior to their enrolment in the study. All clinical and laboratory data were provided to the attending medical team as soon as they were available.

### Data collection

A structured questionnaire was administered to patients or their attendants to collect the following data for each participant: demographics, history of presenting symptoms, and HIV serology status. Examination findings, including vital signs and Glasgow Coma Scale (GCS), and in-hospital mortality were also recorded. The study team followed each patient until discharge or death, but medical management was provided by the admitting medical team.

Laboratory investigations were obtained for each patient at the time of enrolment including complete blood cell concentration (Beckman Coulter, France), random blood glucose (Accu-Chek portable glucose analyzer; Roche diagnostics, Germany), and whole blood lactate (Accutrend Portable Lactate Analyzer; Sports Resource Group, USA). Prior to antibiotic administration, a blood sample was obtained aseptically and 10 mL was inoculated into brain heart infusion broth in duplicates and incubated at 37°C at the MUST Microbiology Laboratory. This laboratory is enrolled in the International Organisation for Standardisation and maintained satisfactory performance throughout the study period. Positive blood cultures were observed for turbidity, a clot or haemolysis; Gram’s stain was done after subculturing on blood agar, MacConkey, and Chocolate agar plates for maximum recovery and morphological identification of the organisms. The plates were incubated at 37°C for 18-24 h aerobically and anaerobically for Chocolate agar. Blood cultures that did not show turbidity were further incubated up to 10 days. Biochemical identification of culture isolates was done according to standard methods. Bacteraemia was defined as bacterial isolation from one or more blood culture bottles. Cultures growing coagulase-negative staphylococci were considered contaminated and not included in the final analyses.

Patients with suspected tuberculosis had sputum obtained for light-emitting diode fluorescent microscopy (LED-FM). For patients whose HIV status was not known or documented at the time of admission, HIV counselling and testing was provided according to national guidelines. Accordingly, a rapid HIV test was done (Determine, Abbott Laboratories, Tokyo, Japan) followed by a confirmatory test for initially positive results (Statpak, Chembio Diagnostic Systems, Inc, Medford, USA). When the results were conflicting, a tie-breaker (Unigold, Trinity Biotech plc, Bray, Ireland) was used.

Malaria blood slides were evaluated via Field’s staining as previously described [12]. For the purposes of quality control, 10% of all slides and all positive slides were double-read by a second experienced and blinded technician. Slides were also double-read in the case of a positive RDT result. In cases of discordant results between two microscopists or between RDT result and microscopy an additional reading was performed by a third microscopist and taken as the definitive microscopy result. A drop of blood was obtained by lancet for malaria RDT according to manufacturer’s directions (SD Bioline Malaria Ag Pf/Pan, Standard Diagnostics, Inc, Korea). This RDT kit uses histidine-rich protein II (HRP-II) for identification of *Plasmodium falciparum* and *Plasmodium* lactate dehydrogenase (pLDH) for identification of *Plasmodium vivax*, *Plasmodium ovale*, and *Plasmodium malariae*.

Blood was also obtained by phlebotomy for PCR testing. Venous blood (100–200 μL) was applied to Whatman FTA Classic Filter paper cards (GE Healthcare Ltd, New Jersey, USA) and left to air dry. These cards deactivate viral DNA/RNA and preserve human and parasite DNA for downstream analyses. DNA extractions were carried out according to the manufacturer's instructions. Briefly, a disc of 1.2 mm in diameter was punched from the centre of each dried blood spotted card and washed three times with Whatman FTA Purification Reagent, and twice with TE buffer. This treated disc was then used directly in subsequent PCR analyses. Nested PCR was performed using the primers and amplification conditions as previously described [13]. Nested PCRs were performed initially to screen for samples containing *Plasmodium* as described. Species of *Plasmodium* in positive samples were determined in separate species-specific nest 2 reactions. The resulting PCR products were visualized on 2% agarose gels, with the presence or absence of a band with each species primer pair indicative of the presence or absence of that species in the initial sample. The laboratory technicians performing PCR were blinded to the results of blood smears and RDTs.

### Statistical analysis

Demographic, clinical and laboratory characteristics of the cohort were summarized. For the primary outcome of interest, the relative contribution of etiologies of sepsis was measured. The causes of sepsis were defined as either definitive or presumptive. Definitive causes were tuberculosis identified by sputum LED-FM, culture-proven bacteraemia, and malaria, as determined by a positive PCR. Presumptive causes of sepsis included malaria (peripheral blood smear or RDT), tuberculosis, community acquired pneumonia, enteric fever, fever of unknown origin and other or unspecified diagnosis. *χ*^2^ testing was used to compare baseline characteristics between those with and without a definitive diagnosis of malaria. Poisson regression models were fitted to determine factors associated with incident hospital mortality, including demographic characteristics, presenting signs, laboratory results, and discharge diagnoses. Finally, multivariable Poisson regression models which included all the characteristics that were significant in univariable models as determined by a p-value <0.25 were performed.

### Ethics statement

Approval was obtained from the institutional review boards of MUST and the University of Virginia prior to the beginning of enrolment in this study.

## Results

### Cohort characteristics

**Figure 1:**
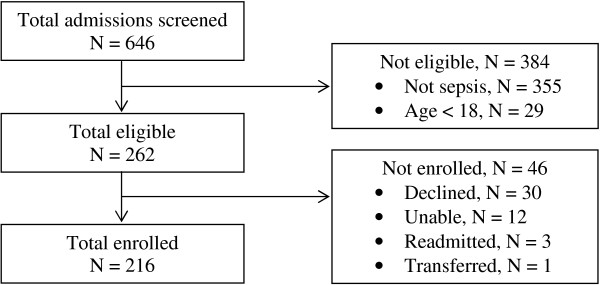
Flow diagram of patient enrolment for the study.

A total of 646 patients were screened and 216 were enrolled (Figure 1). Their median age was 32 years (range 27-42 years) and approximately half were women (Table 1). HIV-infection was present in 56% (122 of 216) of the cohort and 36% (44 of 122) were receiving antiretroviral therapy (Table 2). Of those patients who had a CD4+ T cell test performed, 66% (75 of 113) had a CD4+ T-cell concentration <100 cells/μL. The majority of patients (90%, 194 of 216) complained of fever. Other common complaints included cough (54%, 117 of 216), vomiting (19%, 40 of 216), headache (12%, 26 of 216), and diarrhoea (12%, 25 of 216). Prior to admission, 24% (51 of 216) of patients had received antibiotics and 36% (77 of 216) had received an anti-malarial drug including artemether-lumefantrine (24%, 52 of 216) and quinine (16%, 35 of 216).

**Table 1:** **Summary statistics regarding demographics and clinical characteristics for a cohort of adult patients presenting with sepsis to a regional referral hospital in south**-**western Uganda** (**n** = **216**)

**Characteristic**	**Summary statistic**
**Demographics**	
Age (median, IQR)	32 (27 – 42)
Female, n (%)	110 (51)
Married, n (%)	123 (57)
Educational attainment, n (%)	
None	59 (27)
Primary	121 (56)
Secondary	32 (15)
Tertiary	4 (2)
District of residence, n (%)	
Mbarara	72 (33)
Isingiro	48 (22)
Kiruhura	28 (13)
Other	67 (31)
**Clinical characteristics**	
Past medical history, n (%)	
None	193 (90)
Diabetes mellitus	6 (3)
Kaposi’s Sarcoma	6 (3)
Other	10 (5)
Presenting symptoms, n (%)	
Fever	194 (90)
Cough	117 (54)
Vomiting	40 (19)
Headache	26 (12)
Diarrhoea	25 (12)
Weight Loss	17 (8)
Dyspnea	15 (7)
Nightsweats	13 (6)
Medications prior to admission, n (%)	
Antibiotics	51 (24)
Artemether/lumafantrine	52 (24)
Quinine	35 (16)
Presenting signs, n (%)	
Fever (>38°C)	153 (71)
Tachycardia (100 beats/min)	177 (82)
Tachypnea (>20 breaths/min)	208 (96)
Hypotension (MAP < 65)	62 (29)
Glasgow Coma Score <9	8 (4)
Severe Sepsis	106 (50)

**Table 2:** **Summary statistics regarding test results and hospital treatments for a cohort of adult patients presenting with sepsis to a regional referral hospital in south**-**western Uganda** (**n** = **216**)

**Characteristic**	**Summary statistic**
**Test results**, **n** (% **positive**)	
Anaemia (haemoglobin <9 mg/dl)	93 (43)
Leukocytosis (WBC >12,000/μL)	24 (11)
Thrombocytopenia (platelets < 150/μL)	118 (55)
Lactic Acidosis (lactate >4 mmol/L)	89 (42)
Positive Blood Culture, n (%)	41 (19)
*Staphylococcus aureus*	27 (66)
*Salmonella* spp.	8 (20)
*Streptococcus* spp.	3 (7)
Other/unidentified organism	3 (7)
HIV seropositive with positive blood cultures, n (%)	19 (9)
*Staphylococcus aureus*	17 (89)
*Salmonella* spp.	1 (5)
Other/unidentified organism	1 (5)
HIV seronegative or serostatus unknown with positive blood cultures, n (%)	22 (10)
*Staphylococcus aureus*	10 (45)
*Salmonella* spp.	7 (32)
*Streptococcus* spp.	3 (14)
Other/unidentified organism	2 (9)
HIV seropositive, n (%)	122 (56)
CD4 count <100 cells/μL	75 (66)
ART prior to admission	44 (36)
Cotrimoxazole prior to admission	80 (66)
Malaria test n (% positive)	9 (4)
Positive malaria smear	2 (1)
Positive malaria rapid diagnostic test	6 (3)
Positive malaria PCR	8 (4)
Positive acid-fast bacteria sputum stain, n (%)	24 (11)
**Hospital treatments received**, **n** (%)	
Artemether/lumafantrine	33 (15)
Quinine	45 (21)
Antibiotics	187 (87)
Anti-tuberculosis medicines	16 (7)

### Clinical and laboratory characteristics

The majority of patients were febrile with a temperature >38°C (71%, 153 of 216), tachycardic with a heart rate of ≥100 beats/min (82%, 177 of 216), and tachypneic with a respiratory rate >20 breaths/min (96%, 208 of 216). Half the patients (50%, 106 of 216) had severe sepsis with evidence of end-organ dysfunction. Only 4% (8 of 216) of patients had PCR proven malaria whereas bacteraemia was detected in 19% (41 of 216) of patients and 11% (24 of 216) had tuberculosis as defined by positive sputum LED-FM. The most common presumptive diagnosis at discharge was tuberculosis (28%, 61 of 216) followed by pneumonia (26%, 56 of 216; Table 3). Of those with positive blood cultures, *Staphylococcus aureus* was the most frequently identified pathogen (66%, 27 of 41) followed by *Salmonella* species (20%, 8 of 41) and streptococcal species (7%, 3 of 41). Laboratory evaluation revealed leukocytosis (WBC count >12,000/ml) in 11% (24 of 215), anaemia (haemoglobin <9 mg/dL) in 43% (93 of 216) and thrombocytopenia (platelets <150 cells/μL) in 55% (118 of 215) of participants. Lactic acidosis (lactate >4 mmol/L) was identified in 42% (89 of 211) of patients.

**Table 3:** **Discharge diagnoses and outcomes among patients presenting with sepsis to a regional referral hospital in south**-**western Uganda**

	**Diagnosis at discharge, n (%)**	**Length of stay Median (IQR)**	**Mortality rate %**
Total cohort	216 (100)	5 (3 – 8)	20.0
Definitive diagnosis			
Malaria	8 (3)	4 (3 – 4.5)	12.5
Bacteraemia	41 (19)	5.5 (3 – 8.5)	26.8
Pulmonary tuberculosis	24 (11)	5.5 (2.5 – 13.5)	25.0
Presumptive diagnosis			
Malaria	18 (8)	3 (2 – 5)	11.1
Tuberculosis	61 (28)	7 (4 – 11)	24.6
Enteric fever	19 (9)	4 (4 – 10)	10.5
Community acquired pneumonia	56 (26)	4 (3 – 6)	12.5
Fever of unknown origin	17 (8)	6 (3 – 12)	23.5
Other diagnosis, or unspecified	65 (30)	5 (3 – 9)	21.5

### Malaria diagnosis

Malaria was identified by PCR in eight patients. There were six cases of *P*. *falciparum* and two cases of *P*. *vivax* infection. RDT and blood slide identified five and two of these eight patients respectively. A positive RDT result was 62.5% (95% CI 24.5-91.5) sensitive and 99.5% (95% CI 97.3-100) specific for PCR proven malaria with an 83.3% positive predictive value and 98.6% negative predictive value. The sensitivity and specificity of a positive blood slide for a PCR proven malaria diagnosis was 25% (95% CI 3.2-65.1) and 100% (95% CI 98.2-100) respectively with a positive predictive value of 100% and negative predictive value of 97.2%. A clinical discharge diagnosis of malaria was 62% (95% CI 24.5-91.5) sensitive and 99.5% (95% CI 97.3-100) specific for PCR proven malaria.

Due to the low prevalence of malaria in the cohort, clinical correlates associated with malaria were difficult to determine; however, cough was less common in those with malaria (13%, 1 of 8 *vs* 56%, 116 of 208 in patients without malaria, p = 0.02). A final clinical discharge diagnosis of malaria was given to 50% (4 of 8) of patients with malaria compared to 7% (14 of 208) in those without malaria (p <0.01). Anti-malarial treatment was provided to 63% (5 of 8) of patients with malaria and 23% (48 of 208) of those without malaria (p = 0.01). Antibacterial therapy was provided to 63% (five of eight) and 88% (182 of 208) of patients with and without malaria respectively (p = 0.05).

### Outcomes

The median length of stay was five days (IQR 3-8, range 0-41 days) and in-hospital death occurred in 19% (42 of 216) of the cohort. Among those with a definitive diagnosis of sepsis, mortality was 26.8% in those with bacteraemia, 25% for those with LED-FM sputum positive tuberculosis, and 12.5% in those with malaria. Mortality rates for those with presumptive diagnoses were 24.6% for tuberculosis, 23.5% for fever of unknown origin, 21.5% for an unknown diagnosis, 12.5% for community acquired pneumonia, 11.1% for malaria, and 10.5% for enteric fever (Table 3). In the univariable analysis, GCS <9, severe sepsis, and lactate >4 mmol/L were all significantly associated with in-hospital mortality while fever was associated with survival (p <0.05; Table 4). In the multivariable regression model, the adjusted relative risk (RR) for in-hospital mortality associated with a GCS <9 was 4.8 with a 95% confidence interval (CI) of 1.8-12.8 and p <0.01. Severe sepsis was also independently associated with in-hospital mortality (RR 2.1, 95% CI 1.0-4.1, p = 0.04). Lactate was removed from the multivariable model due to collinearity with severe sepsis. In contradistinction, fever was independently associated with reduced in-hospital mortality (RR 0.5, 95% CI 0.2-0.9, p = 0.02).

**Table 4:** **Incidence of mortality in adult patients presenting to the medical ward at Mbarara Regional Referral Hospital with sepsis** (**Multivariable regression model adjusted for predictors of mortality that met inclusion by a p**-**value** <**0**.**25 in the univariable model**)

**Characteristic**	**Incidence of mortality**	**Incidence rate ratio**	**95% CI**	**p**-**value**	**Adjusted incidence rate ratio**	**95% CI**	**p-value**
**Entire cohort**	19.4	--	--	--			
Age (each 10 years)	--	1.02	0.84 – 1.27	0.78	--		
Male (REF)	24.5						
Female	14.6	0.59	0.32 – 1.11	0.10*	0.57	0.29 – 1.10	0.10
Single (REF)	22.0				--		
Married	17.9	0.81	0.44 – 1.49	0.51			
Educational attainment							
Less than primary (REF)	17.8						
Greater than primary	27.8	1.56	0.77 – 3.18	0.22*	1.45	0.70 – 3.03	0.32
**Clinical characteristics**							
No antibiotics prior (REF)	18.8				--		
Antibiotics prior	21.6	1.15	0.58 – 2.28	0.69			
No fever (REF)	31.8						
Fever	14.4	0.45	0.25 – 0.83	0.01*	0.46	0.24 – 0.88	0.02
No tachycardia (REF)	23.1				--		
Tachycardia	18.6	0.81	0.39 – 1.69	0.57			
No tachypnea (REF)	12.5				--		
Tachypnea	19.7	1.57	0.22 – 11.46	0.65			
No hypotension (REF)	19.5				--		
Hypotension	19.4	0.99	0.51 – 1.94	0.99			
GCS >9 (REF)	17.1						
Glasgow Coma Score <9	75.0	4.39	1.85 – 10.44	<0.01*	4.81	1.80 – 12.8	<0.01
Sepsis (REF)	11.2						
Severe sepsis	27.4	2.44	1.24 – 4.78	0.01*	2.07	1.03 – 4.14	0.04
No aenemia (REF)	21.3				--		
Anaemia	17.2	0.81	0.43 – 1.50	0.50			
No leuckocytosis (REF)	18.3				--		
Leukocytosis	29.2	1.59	0.71 – 3.58	0.26			
No thrombocytopaenia (REF)	17.5				--		
Thrombocytopaenia	21.2	1.21	0.65 – 2.24	0.55			
No lactic acidosis (REF)	13.1				--		
Lactic acidosis	28.1	2.14	1.14 – 4.01	0.02*			
HIV seronegative (REF)	19.2				--		
HIV seropositive	19.7	1.03	0.56 – 1.89	0.93			
**Definitive diagnosis at discharge**							
Negative blood culture (REF)	17.9						
Positive blood culture	26.8	1.50	0.75 – 2.98	0.25*	1.40	0.68 – 2.87	0.36
Negative or no sputum smear for AFB (REF)	18.8				--		
Positive AFB sputum smear	25.0	1.33	0.56 – 3.16	0.51			
No confirmed malaria (REF)	19.1				--		
Confirmed malaria	33.3	1.75	0.43 – 7.24	0.44			
**Clinical diagnosis at discharge**							
Not malaria (REF)	20.2				--		
Malaria	11.1	0.55	0.13 – 2.28	0.41			
Not tuberculosis (REF)	17.4				--		
Tuberculosis	24.6	1.41	0.75 – 2.65	0.28			
Not enteric fever REF)	20.3				--		
Enteric fever	10.5	0.52	0.12 – 2.15	0.36			
Not pneumonia (REF)	21.9				--		
Pneumonia	12.5	0.57	0.24 – 1.29	0.18			
Not fever of unknown origin (REF)	19.1				--		
Fever of unknown origin	23.5	1.23	0.44 – 3.45	0.69			
No other diagnosis (REF)	18.5				--		
Other diagnosis, or unspecified	21.5	1.16	0.61 – 2.21	0.65			

## Discussion

In adult patients presenting to a regional referral hospital in south-western Uganda where malaria transmission is moderate, malaria was an uncommon cause of sepsis. These findings have implications for empirical treatment of sepsis in this region and emphasize the importance of the determination of microbiological diagnoses when treating sepsis. Based on these data, malaria as a sole cause of sepsis should be considered a diagnosis of exclusion among patients presenting with sepsis in similar settings. Accordingly, since the negative predictive value of RDT is so high treatment for malaria should be limited to those in whom malaria diagnosis has been made through a positive RDT.

The low malaria prevalence of approximately 4% in this cohort of adult patients with sepsis contrasts with national Ugandan malaria epidemiology. As a whole, Uganda has a high malaria prevalence (30-50%) which may be increasing [14]. Additionally, annual entomological inoculation rates (EIRs) vary in Uganda from four infective bites per person year in the south-western highlands to >1,500 infective bites per person per year in Apac in the central part of the country [15]. Malaria admissions increased in children in Uganda from 1999 until 2009 but similar data are not available for adults [15]. In contrast to the rest of the country, Mbarara District has had a heterogeneous decrease in malaria in children [11]. Depending on the area within the district and the amount of rainfall, Mbarara has an EIR of 11-100/year and a prevalence of 4-23% [11,14].

The reason for the low malaria prevalence in this cohort compared to the general population may be due to several factors. First, epidemiological data are generally derived from children as adults have improved immunological control of malaria and therefore lower prevalence of infection even in the setting of HIV infection [2]. Severe malaria may occur more often in adults who have waxing and waning immunity to malaria where malaria transmission is unstable and prone to epidemics, such as the highlands of south-western Uganda which does not include Mbarara District [16]. Second, many of the patients (40%) had already received anti-malarial therapy prior to admission. Therefore, any incidental parasitaemia that was present in these patients was likely eradicated prior to admission.

The RDT used in this study employed HRP-II to identify *Plasmodium falciparum* and pLDH to identify non-falciparum species. HRP-II antigen tests are very sensitive and positive results can persist 14 days or more after treatment whereas pLDH provides a negative RDT result within 3 days of treatment [17,18]. There were only 6 positive RDT results in this study. If malaria was a significant cause of sepsis then more positive RDT results would be expected despite any preceding anti-malarial therapy. It cannot be completely ruled out that some of the patients already on treatment could have been falsely-negative for malaria by RDT, but these cases should have been captured by the more sensitive PCR test. Although microscopy and the degree of parasitaemia are more correlated with clinical symptoms, PCR was chosen as the gold-standard in this study in order to apply the most sensitive method to detect malaria parasitaemia. The use of PCR allowed capture of the rare patient that had malaria but had pre-hospital anti-malarial treatment and a subsequently negative malaria slide or RDT result.

A diagnosis of malaria was not associated with increased mortality in this study or in similar studies. Moreover, in contrast to receipt of early antibacterial therapy, receipt of anti-malarial drugs did not improve mortality outcomes in this study or a study of fluid resuscitation in severe sepsis performed in Uganda [19]. In a study performed in a high malaria prevalence area of Tanzania, malaria was not a frequent cause of severe febrile illness in adults [20]. That study used RDTs and blood smears but not PCR to define the presence of malaria parasitaemia. Taken together, the results of this study suggest that parasitaemia occurs very rarely in this patient population and that malaria is infrequently a cause of adult sepsis.

This and prior studies of severe sepsis in Uganda have revealed non-Typhi *Salmonella*, *Streptococcus pneumoniae*, and *Staphylococcus aureus* as common pathogens [5,19,21]. A close temporal association between bacteraemia and malaria has also recently been made clear [22]. There were no cases of dual infection in the current study. However, pre-hospitalization use of antibacterial drugs may have obscured the ability to detect bacteria in blood cultures for some patients in this study. Tuberculosis was a leading discharge diagnosis for this cohort and was associated with approximately 25% mortality. Other studies in SSA have documented *Mycobacterium tuberculosis* (MTb) as a common cause of bloodstream infection in hospitalized patients and these patients have high mortality with 50% of patients dying within 36 days in one study [19,21,23]. Despite these findings, it is rare that antituberculous drugs are included in empiric sepsis therapy in MTb endemic regions such as Uganda. Further investigation into MTb rapid diagnostics and empirical anti-MTb therapy in the setting of sepsis in this region are needed [24]. In this study, patients with altered mental status and severe sepsis were at highest risk of death. This is consistent with prior work from the same institution and others in the region [5,19,21].

There are several limitations of this study. Patients were not evaluated through an entire calendar year and times of high malaria transmission may have been missed. The enrolment period for this study did include one of the rainiest times of year in Mbarara (March-May) which accordingly is a time of high community malaria prevalence [11]. Therefore, it is unlikely that malaria prevalence in adult patients admitted with sepsis would be higher at any other time. However, enrolment of adult septic patients throughout a calendar year might provide additional information on temporal changes in malaria prevalence. Due to the small number of patients with PCR proven malaria in this study, it is not possible to draw definitive conclusions regarding the relative abilities of blood slides and RDTs to diagnose malaria in adult septic patients. These data do suggest that a positive finding by either modality is specific for malaria. Beyond malaria diagnostics, the ability to determine the aetiology of sepsis in these patients was limited. Given the high prevalence of mycobacteraemia that has recently been described in similar patients in SSA it is likely that many of these patients were infected with MTb [19,23]. This conjecture is supported by the large proportion of patients that were assigned a presumptive (28%) or definitive (11%) discharge diagnosis of tuberculosis. Additionally, it was not possible to detect rickettsial diseases, Q-fever, leptospirosis, brucellosis, or histoplasmosis, which have recently been increasingly recognized in the region [25-28].

Despite these limitations, data from this study lend strong support to the notion that malaria is an uncommon cause of sepsis in adult inpatients in south-western Uganda. Based on the findings of this study, the following recommendations are made to practitioners providing care to patients with sepsis in similar settings. First, thorough diagnostic evaluations should be pursued where possible to evaluate for non-malarial causes of sepsis. Second, as recommended by the World Health Organization, anti-malarial therapy should be reserved for patients with a positive RDT or blood slide result to minimize over-prescribing and development of resistance. A bacterial cause of sepsis was identified in ~20% of these patients and it is suggested that empirical treatment of sepsis in this region should include coverage of staphylococci, streptococci, and *Salmonella* species. Unfortunately, the vast majority of patients did not have a confirmed microbiological diagnosis for their sepsis. Further studies are needed to discern the role for empirical anti-MTb therapy in patients presenting with sepsis in high HIV prevalent MTb endemic areas. Improved diagnostic capacity, including development of point-of-care testing, for use in regional referral hospitals in resource-limited settings may improve targeted and empirical therapy for sepsis, reduce development of resistance to anti-malarial agents, and improve outcomes. Finally, given the high prevalence of HIV in this region all patients admitted with sepsis should be tested for HIV according to national guidelines.

## Conclusions

In Mbarara which is located south-western Uganda and where community malaria prevalence is 4-23% malaria was an infrequent cause of sepsis in adults. The ability to detect parasitaemia was maximized through use of PCR technology. Using this sensitive laboratory method, only 8 out of 216 patients were diagnosed with malaria. In contrast, bacteraemia was found in 41 (19%) patients. Additionally, tuberculosis was a definitive diagnosis for 24 (11%) and presumptive diagnosis for 61 (28%) of these patients. When treating adult septic patients in similar regions a thorough evaluation of non-malaria diagnoses with particular emphasis on identifying bacteraemia and tuberculosis is recommended.

## Abbreviations

SSA: Sub-Saharan Africa; HIV: Human immunodeficiency virus; RDT: Rapid diagnostic test; PCR: Polymerase chain reaction; \MRRH: Mbarara Regional Referral Hospital; MUST: Mbarara University of Science and Technology; WBC: White blood cell concentration; GCS: Glasgow coma scale; HRP-II: Histidine-rich protein II; pLDH: *Plasmodium* lactate dehydrogenase; LED-FM: Light-emitting diode fluorescent microscopy; DNA: Deoxyribonucleic acid; RNA: Ribonucleic acid; CD: Cluster of differentiation; IQR: Interquartile range; RR: Relative risk; CI: Confidence interval; EIR: Entomological inoculation rate; MTb: Mycobacterium tuberculosis.

## Competing interests

The authors declare that they have no competing interests.

## Authors’ contributions

MAA helped design the study, enrolled patients, analysed the data, and helped to draft the manuscript. MJS analysed the data and helped to draft the manuscript. DN, AN, MN, and JM-A performed laboratory analyses including malaria PCR and bacterial cultures and helped to draft the manuscript. RM and LAW helped to design the study, analyse the data, and draft the manuscript. YB helped to design the study, supervised the malaria PCR work, helped to analyse the data, and helped to draft the manuscript. CCM conceived of and designed the study, coordinated the study, helped to analyse the data, and drafted the manuscript. All authors read and approved the final manuscript.
